# The complete mitochondrial genome of the leafhopper *Evacanthus acuminatus* (Hemiptera: Cicadellidae: Evacanthinae)

**DOI:** 10.1080/23802359.2019.1687039

**Published:** 2019-11-08

**Authors:** Zhouwei Yuan, Xiao Yang, Can Li, Yuehua Song

**Affiliations:** a School of Karst Science, Guizhou Normal University/State Engineering Technology Institute for Karst Desertification Control, Guiyang, China;; b Guizhou Provincial Key Laboratory for Rare Animal and Economic Insect of the Mountainous Region, College of Biology and Environmental Engineering, Guiyang University, Guiyang, China

**Keywords:** *Evacanthus acuminatus*, leafhopper, mitochondrial genome

## Abstract

The complete mitogenome of *Evacanthus acuminatus* was described in this study. The circular molecule is 14,793 bp in length (GenBank accession no. MK948205), and contains 13 protein-coding genes (PCGs), 22 tRNA genes, two rRNA genes, and an A + T-rich region. The base composition values were A (40.0%), C (11.2%), G (10.0%), and T (38.8%), with an A + T bias (78.8%). ATT, ATG, ATA, TTG were initiation codons and TAA, TAG, T were termination codons. Phylogenetic tree based on concatenated amino acid sequences of 13 PCGs shows that *E. acuminatu* and Idiocerinae clade form sister groups, and all species of different subfamilies congregate with each other, which is consistent with the traditional taxonomy.

The leafhopper *Evacanthus acuminatus* belongs to the subfamily Evacanthinae (Hemiptera: Cicadellidae), which injures plants directly by feeding and damages them indirectly by transmitting plant pathogens (Morris 1971; Hamilton 1996). Many leafhoppers in this subfamily are major pests of several agricultural crops (Nielson 1968). In this study, the adult species of *E. acuminatus* were collected from Fanjing Mountain, Guizhou Province, China (N27°52′, E108°47′). The whole body specimens were placed and stored in ethanol. All specimens examined deposited in the School of Karst Science, Guizhou Normal University, China (GZNU) with an accession number GZNU-ELS-20180001.

The complete mitogenome of *E. acuminatus* is a circular molecule of 14,793 bp in length and reserved in GenBank under Accession no. MK948205. It contains 13 protein-coding genes (PCGs), 22 transfer RNA genes (tRNAs), 2 ribosomal RNA genes (rrnL and rrnS), and an A + T-rich region. The gene order and orientation of *E. acuminatus* were identical with other known Hemiptera species (Zhang et al. 2014; Dai et al. 2018). The base composition values of *E. acuminatus* mitogenome were A (40.0%), C (11.2%), G (10.0%), and T (38.8%), with an A + T bias (78.8%). This mitogenome appeared a negative AT-skew (1.52) and a positive GC-skew (−5.66).

Twenty-four genes were transcribed on the majority strand (N-strand), whereas the others were encoded on the minority strand (J-strand). There are 7 intergenic spacer sequences in a total of 44 bp with length varying from 4 to 12 bp. The largest intergenic spacer is located between A + T-rich region and trnI. A total of 47 bp overlaps had been found at 11 gene junctions and the longest overlap existed between trnS2 and nad1.

Most of 13 PCGs initiate with ATN as the start codons (ATG for cox1, atp6, cox3 and cytb; ATT for cox2, nad1, nad2 and nad6; ATA for nad3, nad4, nad4L, nad5), whereas atp8 used TTG. The conventional termination codons TAA or TAG appear in 10 PCGs, cox2, cox3, and nad5 terminate with a single T residue. The mitogenome includes the complete set of 22 tRNAs, ranging from 60 bp (trnC) to 70 bp (trnK). 22 tRNA genes have the typical cloverleaf structure except for the dihydrouridine arm of trnS1 forms a simple loop, which is identical with previous report in most animal mitogenomes (Wolstenholme 1992). The rrnL gene is located between trnL2 and trnV with length of 1171 bp and A + T content of 80.3%. The rrnS gene is located between trnV and A + T-rich region, with a length of 732 bp and A + T content of 81.3%. The A + T-rich region is located between rrnL and trnI genes is 530 bp in length with an A + T content of 74.7%.

The phylogenetic analysis of *E. acuminatu* and other 13 leafhopper species were performed using the neighbor-joining method based on the amino acid sequences of 13 PCGs. The result shows all species of different subfamilies congregate with each other which is consistent with the traditional taxonomy. Meanwhile, *E. acuminatu* and Idiocerinae clade form sister groups, indicating that they have more closely relationship (Figure 1).

**Figure 1.**
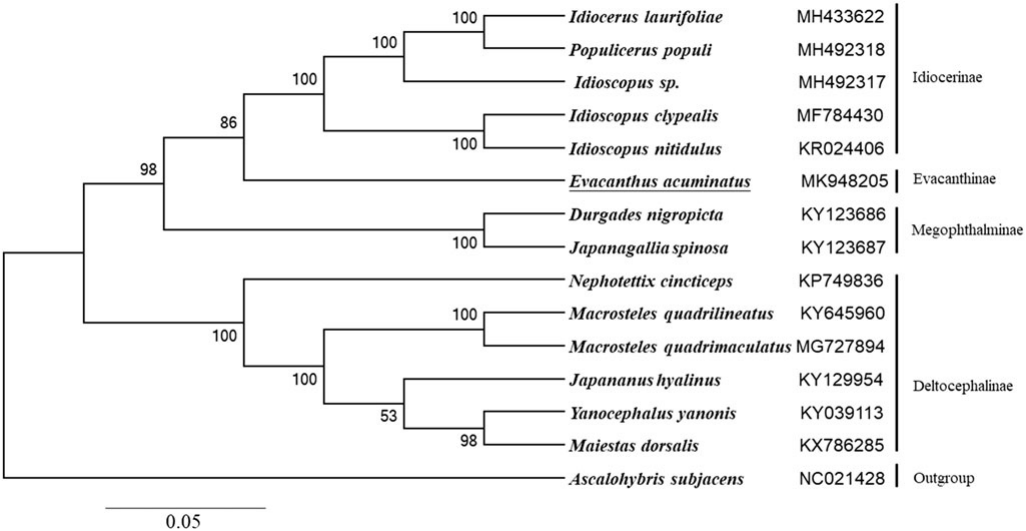
Phylogenetic tree showing the relationship between *E. acuminatus* and 13 other leafhoppers based on the neighbor-joining method. *Ascalohybris subjacens* was used as an outgroup. Leafhopper determined in this study was underlined. GenBank accession numbers of each species were listed in the tree.
